# Metabolic and inflammatory links to rotator cuff tear in hand osteoarthritis: A cross sectional study

**DOI:** 10.1371/journal.pone.0228779

**Published:** 2020-02-10

**Authors:** Young Sun Suh, Hyun-Ok Kim, Yun-Hong Cheon, Mingyo Kim, Rock-Bum Kim, Ki-Soo Park, Hyung Bin Park, Jae-Beom Na, Jin Il Moon, Sang-Il Lee

**Affiliations:** 1 Division of Rheumatology, Department of Internal Medicine, Gyeongsang National University School of Medicine and Gyeongsang National University Changwon Hospital, Changwon, Republic of Korea; 2 Division of Rheumatology, Department of Internal Medicine, Gyeongsang National University School of Medicine and Gyeongsang National University Hospital, Jinju, Republic of Korea; 3 Department of Preventive Medicine, Institute of Health Sciences, Gyeongsang National University School of Medicine, Jinju, Republic of Korea; 4 Department of Orthopedic Surgery, Gyeongsang National University School of Medicine and Gyeongsang National University Changwon Hospital, Changwon, Republic of Korea; 5 Department of Radiology, Gyeongsang National University School of Medicine and Gyeongsang National University Hospital, Jinju, Republic of Korea; 6 Department of Radiology, Gyeongsang National University Changwon Hospital, Changwon, Republic of Korea; Monash University, AUSTRALIA

## Abstract

**Objectives:**

To estimate the prevalence and associated factors of rotator cuff tear (RCT) in patients with hand osteoarthritis (HOA).

**Methods:**

Between June 2013 and December 2015, we recruited 1150 participants in rural area of South Korea. Of the 1150 participants, 307 participants with HOA were analyzed. Plain radiography of both hands, magnetic resonance imaging of both shoulders, and serum levels of high-sensitive C-reactive protein (hsCRP) and high-density lipoprotein (HDL) were obtained for all patients. HOA and RCT were diagnosed by clinical and radiologic findings.

**Results:**

The prevalence of RCT in patients with HOA (192/307, 62.5%) was higher than that in those without HOA (410/827, 49.5%, p<0.001). Among the 307 patients with HOA, the patients with RCT were older, and had higher hsCRP and lower HDL levels than the patients without RCT. Multiple logistic regression analysis confirmed significant associations of age (odds ratio [OR], 1.06; 95% confidence interval [CI], 1.02–1.11), serum hsCRP levels ≥0.6mg/L (OR, 1.68; CI, 1.00–2.80), and low HDL levels (male, <50 mg/dL; female, <40 mg/dL) (OR, 1.93; CI, 1.05–3.56) with RCT in patients with HOA. For patients below 60 years old, the prevalence of RCT was 2.8-fold higher in the low HDL group than normal HDL group (p = 0.048). Finally, the prevalence of RCT was 2.6-fold higher in patients with HOA with both elevated hsCRP and low HDL levels compared with those with neither (p<0.05).

**Conclusions:**

Our findings suggest inflammation and metabolic factors were associated with the prevalence of RCT in HOA patients.

## Introduction

Osteoarthritis (OA) is the leading cause of musculoskeletal morbidity in the elderly, with the hands being the most frequent site of OA development [1,2]. Socioeconomic burden is also high, with direct cost of OA estimated between 1.0% and 2.5% of the gross domestic product in developed countries [3]. It is known that the prevalence of OA increases in the presence of mechanical factors such as hyper-mobility, altered joint mechanical loading, and joint injury, as well as age-related degenerative change [4–7]. However, inflammation and metabolic factors such as dyslipidaemia, have emerged as important causes in recent studies [8–10].

Rotator cuff tear (RCT) is one of the most common shoulder disorders [11]. Causes include degenerative factors such as aging, mechanical factors, and anatomic factors; similar to OA [12]. Recently, inflammation and metabolic factors were also considered as crucial causes of RCT [13]. Surgical treatment is eventually required for RCT when conventional treatment fails. An estimated 250,000 to >500,000 repairs are performed annually, and in the United States the mean costs for surgical treatments were $15,063 per episode [14]. Thus, early diagnosis and proper treatment of RCT are important in minimizing the burden of RCT.

Both diseases are frequently accompanied and shared etiology [15,16]. According to shared etiology, inflammation and dyslipidemia affect the prevalence of rotator cuff tear in hand osteoarthritis. However, no previous study investigated the relationship between RCT and HOA. Therefore, this study was conducted to estimate the prevalence of RCT and evaluate the factors associated with the prevalence of RCT in patients with HOA.

## Methods

### Research subjects

This cross-sectional study used the HOA subgroup data of the NAMGARAM cohort. The NAMGARAM cohort is made to determine the prevalence and risk factors of upper extremity musculoskeletal disorders in a rural province of South Korea. From June 2013 to August 2015, six villages randomly selected from villages in Gyeongnam Province. Participants consisted of those who were over 40 years old and who agreed to take part in the study among the residents living in Jinju, Sichuan, Changwon, Haman, Hamyang and Sancheong of South Korea. Residents who did not agree with the study were excluded. A total of 1150 people were enrolled in this study. For the study, the participants visited Gyeongsang National University Hospital and conducted a comprehensive examination. Of the 1150 participants, 307 participants with HOA were analyzed. All participants were required to provide written informed consent. The study was approved by the Institutional Review Board of the Gyeongsang National University Hospital (GNUH 2015-02-001). This study was supported by a grant of the Centre for Farmer’s Safety and Health, Ministry of Agriculture, Food and Rural Affairs, Republic of Korea.

### Measurements

Study participants answered the questionnaires, and underwent physical examinations, blood tests, plain radiographies of both hands and shoulders, and magnetic resonance imaging (MRI) of both shoulders. The one-on-one survey was conducted by nurses who were informed about the objective of this study and who were trained in data collection procedures, and it took approximately 30 minutes to complete the questionnaire. The survey included information on socio-demographic variables (age, sex, level of education, smoking, marriage, underlying diseases, height, weight, body mass index [BMI], waist circumference, blood pressure, work hours per day, and total work period); the Patient Health Questionnaire-2 (PHQ-2) [17], a survey tool measuring the degree of depressive symptoms; and the Korean version of the Australian/Canadian (AUSCAN) Osteoarthritis Hand Index, which evaluates pain and functional limitations of HOA [18]. The total working load was defined as the multiplication of the mean working hours per day and the total working period.

Cholesterol tests (total cholesterol, high-density lipoprotein cholesterol [HDL-C], triglycerides [TG], and low-density lipoprotein cholesterol [LDL]), glycated haemoglobin (HbA1c), and high-sensitive C-reactive protein (hsCRP) were performed. Blood tests were performed on an empty stomach for 8 hours to ensure the precise measurement of fasting plasma glucose and HDL, which correspond to items of metabolic syndrome in the questionnaire. The biospecimens and data used for this study were provided by the Gyeongsang National University Hospital, a member of the Korea Biobank Network.

Anterior–posterior plain radiographs of both hands and shoulders were obtained from all participants. The interpretation of these radiographs was performed by a musculoskeletal radiology specialist (20 years of experience in radiographic evaluation) and a rheumatology specialist (10 years of experience in radiographic evaluation). Both readers were blinded to subject history. The second to fifth distal interphalangeal (DIP), proximal interphalangeal (PIP), first to fifth metacarpophalangeal (MCP), thumb interphalangeal (IP), and first carpometacarpal (CMC) joints for each hand were graded for radiographic HOA using the modified Kellgren–Lawrence (KL) scale to assess the existence and severity of osteophytes, joint space narrowing (JSN), sclerosis, and erosion. The modified KL scale was graded from 0 to 4, where 0 is no OA; 1 is questionable osteophytes (OPs) and/or JSN; 2 is definite small OPs and/or mild JSN; 3 is moderate OPs and/or moderate JSN, sclerosis, and possible presence of erosions; and 4 is large OPs and/or severe JSN, sclerosis, and possible presence of erosions [19–21]. The diagnosis of HOA was based on the American College of Rheumatology 1990 guideline, and defined as pain or stiffness on most days of the prior month in addition to three of the following criteria: bony swelling of ≥2 of the 10 selected joints (bilateral 2nd and 3rd DIPs, 2nd and 3rd PIPs, and 1st CMC joint), bony swelling of ≥2 DIP joints, <3 swollen MCP joints, and deformity of ≥1 of the 10 selected joints [22]. Radiological HOA was defined as a case wherein the result of plain radiography was determined to be higher than KL grade 2 of at least one joint [19]. The total number of affected joints and the sum of KL grades of all the affected joints were evaluated while assessing the OA burden of radiological HOA; OA burden was speculated to increase in proportion with the evaluated value. Erosive HOA was defined radiographically by subchondral erosion, cortical destruction and subsequent reparative change, which may include bony ankyloses [23]. The Cohen's kappa correlation coefficient for agreement between the two readers was 0.824 for scoring total KL grades, suggesting excellent level of agreement.

3.0 tesla (T) MRI equipment (Ingenia; Philips Medical Systems, Eindhoven, Netherlands) was used to obtain MRI scans, which included axial, sagittal, and coronal T2-weighted images (repetition time [TR]/echo time [TE] = 2800/60); coronal T1-weighted images (TR/TE = 500/20); and coronal fat-saturated fast spin-echo images. The field of view was 16 cm, the data matrix size was 448 × 448, and the slice thickness was 3 mm without gaps. RCT was diagnosed by the involved shoulder pain and the results of the MRI images, included from partial to complete tear of at least one tendon based on the interpretations of two musculoskeletal radiology specialists (10 years and 20 years of experience in radiographic evaluation). Both readers were blinded to subject history. The Cohen's kappa correlation coefficient for agreement between the two readers was 0.839 for MRI, suggesting excellent level of agreement.

### Statistical analysis

SPSS for Windows (version 20.0, SPSS Inc., Chicago, IL, USA) was used for statistical analysis. The HOA with RCT group and without RCT group were compared to evaluate associated factors of RCT prevalence in HOA patients. Differences between two groups were evaluated using student’s t test or Mann-Whitney test for continuous variables and chi-square test for categorical variables. Cohen's kappa correlation for agreement was used for evaluating agreements in the interpretation of MRI and scoring radiographic KL grades. To identify factors independently affecting the prevalence of RCT in people with HOA, logistic regression analysis was performed, including the variables with p-values <0.2 in initial univariate analysis and variables expected to be relevant in previous studies. P-values <0.05 were defined as statistically significant in all analyses.

## Results

### The prevalence of RCT in HOA

Of the 307 participants with HOA, 192 (62.5%) had RCT, whereas among the other 827 participants without HOA, 417 (49.5%) had RCT; thus indicating that the prevalence of RCT was higher in patients with HOA than in those without (p<0.001) (S1 Fig). After adjusting confounding factors, HOA tended to affect RCT prevalence (OR 1.16 C.I 0.79–1.71) and total sum of KL grades, which meant the severity of radiographic HOA was associated with RCT prevalence (OR 1.02 C.I 1.00–1.05) with statistical significance (S1 Table).

### Baseline characteristics and factors associated with the prevalence of RCT in HOA

The basic characteristics of the groups with HOA with and without RCT were compared to identify any differences. HOA with RCT group were older (62.69±7.04 vs. 59.10±7.66, p<0.001) than HOA without RCT group. In HOA with RCT group, the total sum of KL grades (9.0 [3.0–18.0] vs. 6.0 [1.0–14.0], p = 0.027) were high, resulting in higher HOA burden compared with HOA without RCT group. In addition, patients with HOA with RCT showed higher serum hsCRP levels (1.51±3.78 vs. 0.67±0.70, p = 0.004), and a higher ratio in men and women with HDL levels <40 mg/dL and <50 mg/dL was also observed compared with those without RCT (27.6% vs. 15.7%, p = 0.018). No differences were found in the total working period (years) and total working load, which was defined as the multiplication of the mean working hours per day by the total working period (Table 1).

**Table 1:** Comparison of the participants with rotator cuff tear and those without rotator cuff tear in hand osteoarthritis (n = 307).

	HOA (+) RCT(+) (n = 192)	HOA (+) RCT(-) (n = 115)	p
Age (mean±SD, years)	62.69±7.04	59.10±7.66	<0.001
Sex			0.249
Female	129 (67.2)	85 (73.9)	
Male	63 (32.8)	30 (26.1)	
Level of education			0.059
≤Elementary school level	95 (49.5)	44 (38.3)	
Middle school level	48 (25.0)	37 (32.2)	
≥High school level	49 (25.5)	34 (29.5)	
Marriage (yes)	168 (87.5)	103 (89.6)	0.715
Smoking (yes)	38 (19.8)	26 (22.8)	0.562
Depressive symptom (yes)	29 (15.1)	14 (12.2)	0.502
Total working period (years)	32.82±14.09	31.00±12.87	0.206
Total working load (hours * years)^a^	255.24±130.11	249.00±117.43	0.666
Total number of involved joints	3.0 (0.0–6.0)	2.0 (0.0–5.0)	0.063
Total sum of KL grades	9.0 (3.0–18.0)	6.0 (1.0–14.0)	0.027
AUSCAN			
Pain (0–500)	136.56±119.12	110.12±106.63	0.061
Stiffness (0–100)	32.02±28.30	28.77±26.10	0.441
Function (0–900)	137.67±147.15	135.40±152.48	0.701
Total score (0–1500)	339.22±283.05	308.22±273.35	0.397
hsCRP (mg/L)	1.51±3.78	0.67±0.70	0.004
HDL (mg/dL)	55.66±15.46	60.48±12.45	0.001
HbA 1c (%)	5.93±0.66	5.92±0.73	0.938
BMI (kg/m^2^)	24.63±2.80	24.27±2.66	0.239
Metabolic syndrome (yes)	59 (30.7)	24 (20.9)	0.064
Waist circumference (M≥90 cm, F≥80 cm)	116 (60.4)	67 (58.3)	0.720
BP≥130/85	106 (55.2)	55 (47.8)	0.238
HDL (M<40, F<50 [mg/dL])	53 (27.6)	18 (15.7)	0.018
TG≥150 mg/dL	58 (30.2)	27 (23.5)	0.236
Fasting glucose≥110 mg/dL	33 (17.2)	19 (16.5)	0.999
Inflammatory HOA	37 (19.3)	20 (17.4)	0.762

Categorical variables are the number (percentage).

^a^ Total working load defined as the multiplication of the mean working hours per day by the total working period.

Abbreviations: N, number of patients; RCT, rotator cuff tear; SD, standard deviation; KL, Kellgren–Lawrence; AUSCAN, Australian/Canadian Osteoarthritis Hand Index; hsCRP, high-sensitive C-reactive protein; BMI, body mass index; M, male; F, female; BP, blood pressure; HDL, high-density lipoprotein; TG, triglyceride; HOA, hand osteoarthritis

Logistic regression analysis was performed to examine whether metabolic and inflammatory factors were significantly associated with the prevalence of RCT in the HOA group, with the models being adjusted for other factors that were significant in univariate analyses. Serum hsCRP determined the cut-off value (0.6 mg/L) through regression tree analysis, and HDL used known metabolic syndrome criteria (male<40mg/dL, female<50mg/dL). Age (odds ratio [OR], 1.06; confidence interval [CI], 1.02–1.11); serum hsCRP ≥0.6 mg/L (OR, 1.68; CI, 1. 004–2.80), an inflammatory factor; and serum HDL (male<40 mg/dL, female<50 mg/dL) (OR, 1.93; CI, 1.05–3.56), one of the metabolic factors, were significantly associated with the prevalence of RCT (Table 2).

**Table 2:** Risk factors of rotator cuff tear in hand osteoarthritis using multivariate logistic regression analysis.

	Adjusted OR (95% CI)	p value
Age	1.06 (1.02–1.11)	0.003
Sex (male)	1.66 (0.93–2.94)	0.086
Level of education (≥High school level)	0.87 (0.44–1.75)	0.704
Total sum of KL grades	1.00 (0.98–1.03)	0.780
hsCRP (≥0.6 mg/L)	1.68 (1.00–2.80)	0.049
Low HDL (M<40, F<50 [mg/dL])	1.93 (1.05–3.56)	0.034
BMI	1.08 (0.98–1.19)	0.102

Abbreviation: KL grades, Kellgren–Lawrence grades; hsCRP, high-sensitive C-reactive protein; HDL, high-density lipoprotein cholesterol; M, male; F, female; BMI, body mass index.

### Influence of hsCRP and HDL on RCT prevalence in HOA based on age

Patients with HOA were divided into two groups (≥0.6 mg/L and <0.6 mg/L), and a stratified analysis on the risk of RCT prevalence based on age was performed. There was a trend that hsCRP values above 0.6 mg/L group showed a higher prevalence of RCT compared to the group with hsCRP values below 0.6 mg/L at age under 60 (OR 1.66 C.I 0.73–3.79) and 60s (OR 1.94 C.I 0.86–4.36), but there was no statistical significance (Table 3). When a stratified analysis by age was performed among patients stratified by normal and low HDL values (male<40 mg/dL, female<50 mg/dL), the prevalence of RCT in the low HDL group was 2.8-fold greater than that in the control group among people younger than 60 years (OR, 2.82; CI, 1.01–7.91) with a statistical significance (Table 4).

**Table 3:** Association between high-sensitive C-reactive protein and rotator cuff tear in relation to age.

	Prevalence for RCT (%)			
Age	hsCRP<0.6 mg/L	hsCRP≥0.6 mg/L	Adjusted OR* (95% CI)	*p* value
<60 years	30/70 (42.9)	38/55 (69.1)	1.66 (0.73–3.79)	0.229
60–69 years	45/72 (62.5)	40/57 (70.2)	1.94 (0.86–4.36)	0.109
≥70 years	12/17 (70.6)	24/32 (75.0)	0.77 (0.11–5.40)	0.796
Total group	87/159 (54.7)	102/144 (70.8)	1.68 (1.00–2.80)	0.049

* Adjusted for age, sex, level of education, HDL, total sum of Kellgren–Lawrence grades and BMI.

Abbreviation: RCT, rotator cuff tear; HDL, high-density lipoprotein cholesterol; hsCRP, high-sensitive C-reactive protein; OR, odds ratio; CI, confidence interval

**Table 4:** Association between high-density lipoprotein cholesterol and rotator cuff tear in relation to age.

	Prevalence of RCT (%)			
Age	Normal HDL	Low HDL	Adjusted OR* (95% CI)	*p* value
<60 years	47/99 (47.5)	22/28 (78.6)	2.82 (1.01–7.91)	0.048
60–69 years	64/98 (65.3)	23/33 (69.7)	0.92 (0.38–2.23)	0.852
≥70 years	28/39 (71.8)	8/10 (80.0)	12.16 (0.91–162.82)	0.059
Total group	139/236 (58.9)	53/71 (74.6)	1.93 (1.05–3.56)	0.034

* Adjusted for age, sex, level of education, hsCRP, total sum of Kellgren–Lawrence grades and BMI.

Abbreviation: RCT, rotator cuff tear; HDL, high-density lipoprotein cholesterol; M, male; F, female; OR, odds ratio; CI, confidence interval; hsCRP, high-sensitive C-reactive protein

### Influence of the coexistence of inflammatory and metabolic factors on RCT prevalence in HOA

**Fig 1:**
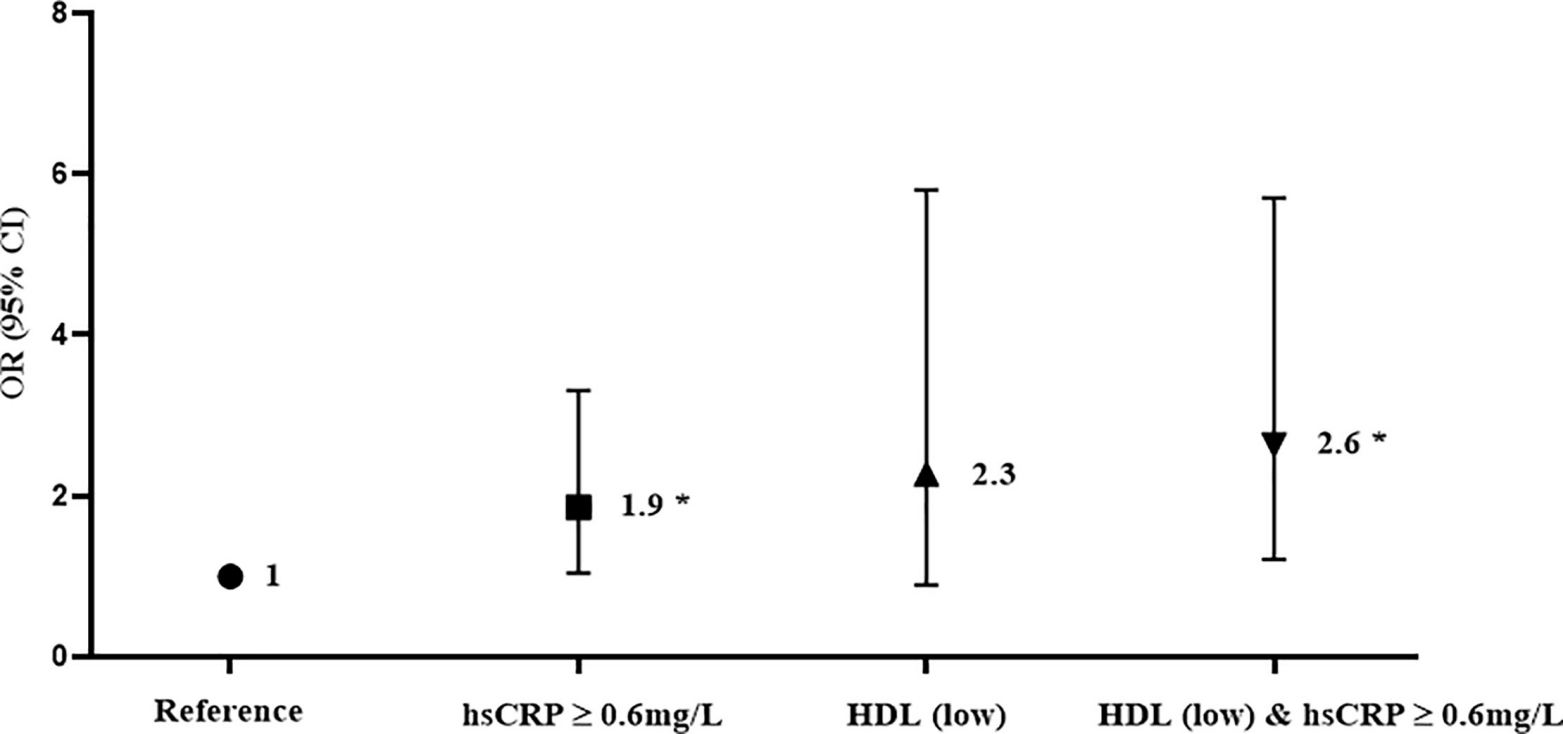
Association between the concurrent presence of inflammatory and metabolic factors and the development of rotator cuff tear in hand osteoarthritis. Adjusted for sex, age, level of education, body mass index and total sum of Kellgren–Lawrence grades. Abbreviation: RCT, rotator cuff tear; hsCRP, high-sensitive C-reactive protein; HDL, high-density lipoprotein cholesterol; M, male; F, female; OR, odds ratio; CI, confidence interval * p<0.05.

To investigate the influence of the coexistence of inflammatory and metabolic factors on RCT prevalence, OR values were calculated for 4 groups; participants with normal HDL and hsCRP values <0.6 mg/L (reference), normal HDL and hsCRP values ≥0.6 mg/L (group 1), low HDL and hsCRP values <0.6 mg/L (group 2), and low HDL and hsCRP values ≥0.6 mg/L (group 3). Compared with the reference group (68/137, 49.6%), participants with hsCRP values ≥0.6 mg/L showed a 1.9-fold higher ratio of RCT prevalence (69/99, 69.7%), those with low HDL showed a 2.3-fold increase (18/26, 69.2%), and those with hsCRP values ≥0.6 mg/L and low HDL showed a 2.6-fold increase (34/46, 73.9%). This indicated that HOA patients with high hsCRP values, which is an inflammatory factor, and with low HDL, which is a metabolic factor, more often also had RCT (Fig 1).

## Discussion

In this study, we aimed to identify the association between RCT and HOA. RCT was prevalent in patients with HOA compared with that in those without HOA. Even after adjusting confounding factors, HOA and RCT may relate, and especially severe radiographic HOA is significantly related to RCT. In addition to age, serum hsCRP level, an inflammatory factor, and serum HDL-C level, a metabolic factor, were associated with the development of RCT in patients with HOA. In particular, the association of metabolic factors with RCT was stronger in participants younger than 60 years. In addition, increased RCT prevalence was associated with presence of both inflammatory and metabolic factors. We conclude that a strong link exists between inflammatory and metabolic factors with the prevalence of RCT in patients with HOA.

To our knowledge, this study is the first report to report the prevalence of RCT in patients with HOA. Previous studies reported that the prevalence of RCT in the general population ranges from 3% to 39% [24,25]. This variation in the prevalence of RCT was influenced by the different methods that were used to diagnose RCT, and which included physical examination, arthrography, ultrasonography, and MRI [26–28]. However, the prevalence of RCT among patients with HOA has never been investigated. Our study showed a high prevalence of RCT in patients with HOA compared to those without HOA. The results in this study are more reliable because we used MRI to improve the accuracy of the diagnosis of RCT. Thus, patients with HOA are believed to need careful examination for possible RCT occurrence.

The relationship between these two diseases can be explained in three ways. First, both HOA and RCT are age-related degenerative diseases with the prevalence and severity of OA being associated with aging [29]. Similarly, RCT has a positive correlation with age and also comes from age-related histological and molecular changes [30,31]. Secondly, inflammatory reactions are involved in both diseases. Actually, the levels of pro-inflammatory cytokines, such as interleukin-1 and -6 and tumour necrosis factor-α are all generally increased in both diseases [32–35]. Thirdly, metabolic factors, such as diabetes, obesity, and dyslipidaemia link to the development of both diseases [36–38]. Our study also demonstrated that age and inflammatory and metabolic factors serve as important common factors involved in the occurrence both diseases. This result suggests the presence of a common mechanism between the two diseases.

Other studies have identified that inflammation is associated with OA. Bos et al. showed that high basal hsCRP levels may influence OA onset [39]. A prospective cohort study showed that CRP was associated with disease progression in patients with OA [40]. A recent meta-analysis demonstrated the relationship between serum CRP levels and OA [41]. Similarly, evidence of inflammation in the development of RCT has been reported, with high concentrations of inflammatory cytokines in the bursal tissue of RCT patients [42,43]. However, no study has demonstrated the important role of inflammation in the correlation between RCT and HOA. Our study showed that serum hsCRP is significantly associated with the prevalence of RCT in patients with HOA, and the prevalence of RCT increased 1.7-fold in patients with HOA with hsCRP≥0.6 mg/L. Thus, we suggest that low-grade systemic inflammation plays a crucial part in the occurrence of RCT in HOA and must be controlled properly.

Recent studies have shown that metabolic factors, such as diabetes, hypertension, high TG levels, and the total cholesterol to HDL ratio, were associated with the occurrence of OA [44–47]. Several studies have also analysed the correlation between metabolic factors and RCT. A prospective study of patients with RCT showed higher levels of total cholesterol, TG, and LDL, and lower levels of HDL in patients compared to the control group [48]. Kim et al. showed that hyperlipidaemia may be a factor that adversely affects the treatment of RCT [49]. However, no study investigated the effect of metabolic factors on the occurrence RCT in patients with HOA. This study was the first study to demonstrate that lower HDL levels are the associated factor of RCT in patients with HOA; particularly in those younger than 60 years. In addition, we found that patients with HOA with lower HDL levels and higher hsCRP levels were more susceptible to RCT than patients with HOA with normal HDL levels and lower hsCRP levels. Therefore, our study revealed that metabolic and inflammatory factors were important to predict RCT in patients with HOA; these factors must be well controlled during HOA treatment.

However, this study is a cross-sectional study, and thus the causal relationship between inflammatory or metabolic factors and RCT could not be determined. Therefore, HOA patients with RCT may not be able to exercise well and may be accompanied by metabolic problems (such as obesity and dyslipidemia), and low grade inflammation as a result. A further longitudinal study is needed to clarify the causes of RCT in patients with HOA. Secondly, as the subject of this study was limited to those who agreed to study in some regions, there could be selection bias. Third, we found that RCT is prevalent in HOA patients compared to without HOA. However, the participants without HOA are not a matched population, so comparison with a calibrated control is needed. Lastly, our study was based on only clinical HOA patients. In addition to clinical HOA, further analysis of radiological HOA is needed.

In conclusion, the higher serum hsCRP level, a representative of inflammatory markers, and low HDL level, one of the metabolic factors, are more important associated factors for RCT in individuals with HOA; particularly for patients who have both factors. Therefore, patients with HOA with elevated CRP levels and/or low HDL levels need to be examined to determine whether RCT occurred together with HOA.

## Supporting information

S1 Fig Flowchart of participants for analysis. Abbreviations: MRI, magnetic resonance imaging; N, number of patients; OA, osteoarthritis; RCT, rotator cuff tear
(JPG)

S1 Table Factors associated with the prevalence of rotator cuff tear in all participants: the association between rotator cuff tear and hand osteoarthritis. (DOCX)
