# Supplementary figures and images for: Metabolic and inflammatory links to rotator cuff tear in hand osteoarthritis: A cross sectional study
Source: PLoS One. 2020 Feb 10;15(2):e0228779. doi: 10.1371/journal.pone.0228779 (PMC7010271; doi:10.1371/journal.pone.0228779)

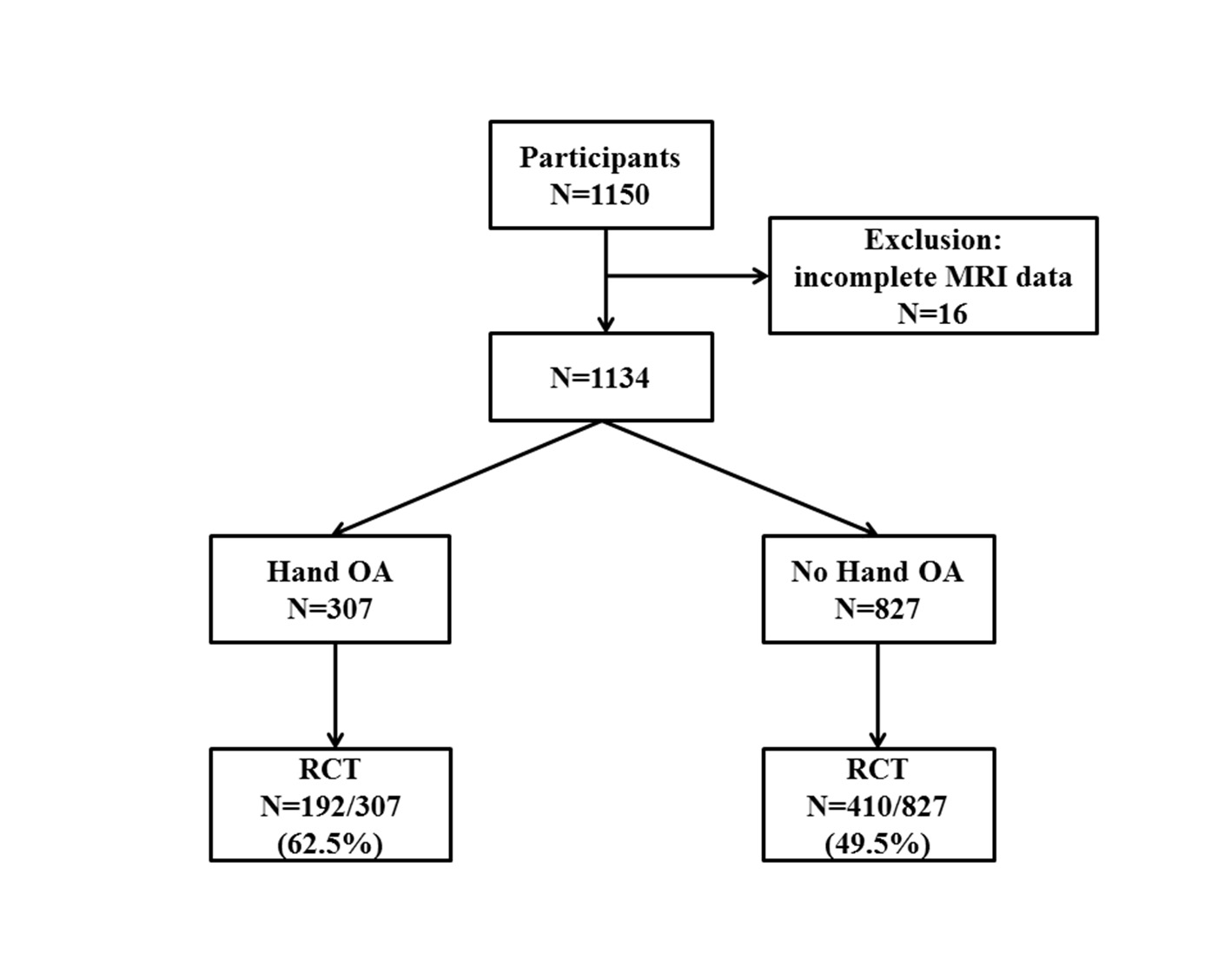

Supplement: S1 Fig — Abbreviations: MRI, magnetic resonance imaging; N, number of patients; OA, osteoarthritis; RCT, rotator cuff tear (JPG) [file pone.0228779.s001.jpg]
