# Supplementary material for: Metabolic and inflammatory links to rotator cuff tear in hand osteoarthritis: A cross sectional study
Source: PLoS One. 2020 Feb 10;15(2):e0228779. doi: 10.1371/journal.pone.0228779 (PMC7010271; doi:10.1371/journal.pone.0228779)
Supplement: S1 Table — (DOCX) [file pone.0228779.s002.docx]

**S1 Table. Factors associated with the prevalence of rotator cuff tear in all participants: the association between rotator cuff tear and hand osteoarthritis**

|  | **crude OR (95%CI)** | **Adjusted OR* (95%CI)** | **p value** |
| --- | --- | --- | --- |
| Hand OA | 1.41 (1.01 - 1.98) | 1.16 (0.79 - 1.71) | 0.447 |
| Total sum of KL grades | 1.05 (1.03 - 1.07) | 1.02 (1.00 - 1.05) | 0.038 |

* Adjusted for sex, age, level of education, body mass index, total working load, hsCRP, and HDL.

Abbreviations: OR, odds ratio; CI, confidence interval; OA, osteoarthritis; KL grades, Kellgren–Lawrence grades; hsCRP, high-sensitive C-reactive protein; HDL, high-density lipoprotein cholesterol.
